# Potassium affects the association between dietary intake of vitamin C and NAFLD among adults in the United States

**DOI:** 10.1371/journal.pone.0295986

**Published:** 2024-04-18

**Authors:** Xu-Hua Liu, Hao-Kai Chen, Jing Luo, Xiang-Ping He, Wan-Lin Zhang, Yue Chen, Xiu-Juan Li, Qi-Wen Lan, Xiao-Man Ma, Xu-Guang Guo

**Affiliations:** 1 Department of Clinical Laboratory Medicine, Guangdong Provincial Key Laboratory of Major Obstetric Diseases, Guangdong Provincial Clinical Research Center for Obstetrics and Gynecology, The Third Affiliated Hospital of Guangzhou Medical University, Guangzhou, China; 2 Department of Clinical Medicine, The Third Clinical School of Guangzhou Medical University, Guangzhou, China; 3 Department of Chinese and Western Clinical Medicine, The Chinese and Western Clinical School of Guangzhou Medical University, Guangzhou, Guangdong, China; 4 Department of Clinical Medicine, The First Clinical School of Guangzhou Medical University, Guangzhou, Guangdong, China; 5 Department of Anesthesia, The Second Clinical School of Guangzhou Medical University, Guangzhou, Guangdong, China; 6 Department of Medical Imageology, The Second Clinical School of Guangzhou Medical University, Guangzhou, Guangdong, China; 7 Guangzhou Key Laboratory for Clinical Rapid Diagnosis and Early Warning of Infectious Diseases, King Med School of Laboratory Medicine, Guangzhou Medical University, Guangzhou, Guangdong, China; Texas A&M University College Station, UNITED STATES

## Abstract

**Introduction:**

Although the association between nonalcoholic fatty liver disease (NAFLD) and vitamin C has been well studied, the effects of dietary potassium intake on this relationship are still unclear. Thus, this study aimed to determine the effects of dietary potassium intake on the association between vitamin C and NAFLD.

**Methods:**

We performed a cross-sectional learn about with 9443 contributors the usage of 2007–2018 NHANES data. Multiple logistic regression evaluation has been utilized to check out the affiliation of dietary vitamin C intake with NAFLD and advanced hepatic fibrosis (AHF). Subsequently, we plotted a smoothed match curve to visualize the association. Especially, the analysis of AHF was conducted among the NAFLD population. In addition, stratified evaluation used to be developed primarily based on demographic variables to verify the steadiness of the results. Effect amendment by way of dietary potassium intake used to be assessed via interplay checks between vitamin C and NAFLD in the multivariable linear regression.

**Results:**

In this cross-sectional study, we found that vitamin C was negatively related to NAFLD and AHF. The relationship between vitamin C and NAFLD was different in the low, middle and high potassium intake groups. Furthermore, potassium intake significantly modified the negative relationship between vitamin C and NAFLD in most of the models.

**Conclusion:**

Our research showed that potassium and vitamin C have an interactive effect in reducing NAFLD, which may have great importance for clinical medication.

## 1. Introduction

Nonalcoholic fatty liver disease (NAFLD) is now recognized as the most ubiquitous liver disease worldwide [1, 2] and is characterized by extensive liver changes that may progress to liver fibrosis and cirrhosis [2]. Twenty-five percent of adults worldwide have NAFLD [1], and the prevalence of NAFLD is now on the rise as obesity rates increase worldwide [3]; NAFLD has also been noted as a familiar etiology of liver disease [1]. In addition to type 2 diabetes, cardiovascular disease, chronic kidney disease, and extrahepatic malignancies, NAFLD is strongly associated with cardiopulmonary disease [4]. It has been shown in a number of animal studies that increased levels of reactive oxygen species and lipid peroxide products are the main cause of liver injury in NAFLD [5], and significantly elevated levels of lipid peroxides have been found in patients with NAFLD [6], indicating that free radical-mediated damage leading to cellular injury is common in this disease.

Vitamin C (VC) is a powerful antioxidant that scavenges free radicals, acts as a reducing agent in a variety of enzymatic reactions [7], and regulates the gut microbial community [8]. After collecting nationally representative data from the 2017–2018 National Health and Nutrition Survey, Zhi-Qin Xie obtained results indicating a negative association between serum VC levels and NAFLD [9]. Jie Wei and his colleagues also found a negative association between dietary vitamin C intake and NAFLD in a study of middle-aged and older adults [10]. A randomized clinical trial also showed that oral vitamin C improved liver health and glucose metabolism in patients with NAFLD [11]. Therefore, it is important to properly understand the correlation between vitamin C and NAFLD.

It’s researched that potassium deficiency may lead to various metabolic disorders, which is a potential risk factor for NAFLD [12–15]. Potassium ions (K) are essential minerals for human health and affect organs such as the heart, kidney and bone [16]. Previous research has shown that patients with NAFLD have lower blood potassium levels than healthy individuals [15]. Potassium may contribute to the onset of NAFLD by impairing insulin production [12, 13]. A cross-sectional study of middle-aged and elderly people in China found that hypokalemia was significantly related to the prevalence of NAFLD [17].

However, the association between VC and NAFLD in US adults with high levels of K exposure is unclear, and there are no clear clinical studies assessing the effect of K on the relationship between VC and NAFLD. In this cross-sectional study, we plan to examine the association between VC and NAFLD and to discover the potential interaction of K in this relationship.

## 2. Materials and methods

### 2.1 Data sources and study design

Data for the current study were collected from NHANES 2007–2018. NHANES is a cross-sectional, nationally representative study created to gather information on diet and health for a noninstitutionalized population in the United States. Demographic, socioeconomic, and health-related information was obtained through questionnaires and physical and laboratory examinations. Dietary assessment was obtained through a 24-h dietary recall. Participants underwent a physical examination, and blood samples were collected at the Flow Examination Center (MEC). A study on dietary intake of vitamin C and NAFLD used data from NHANES 2007–2018 and enrolled a total of 59842 participants.

**Fig 1:**
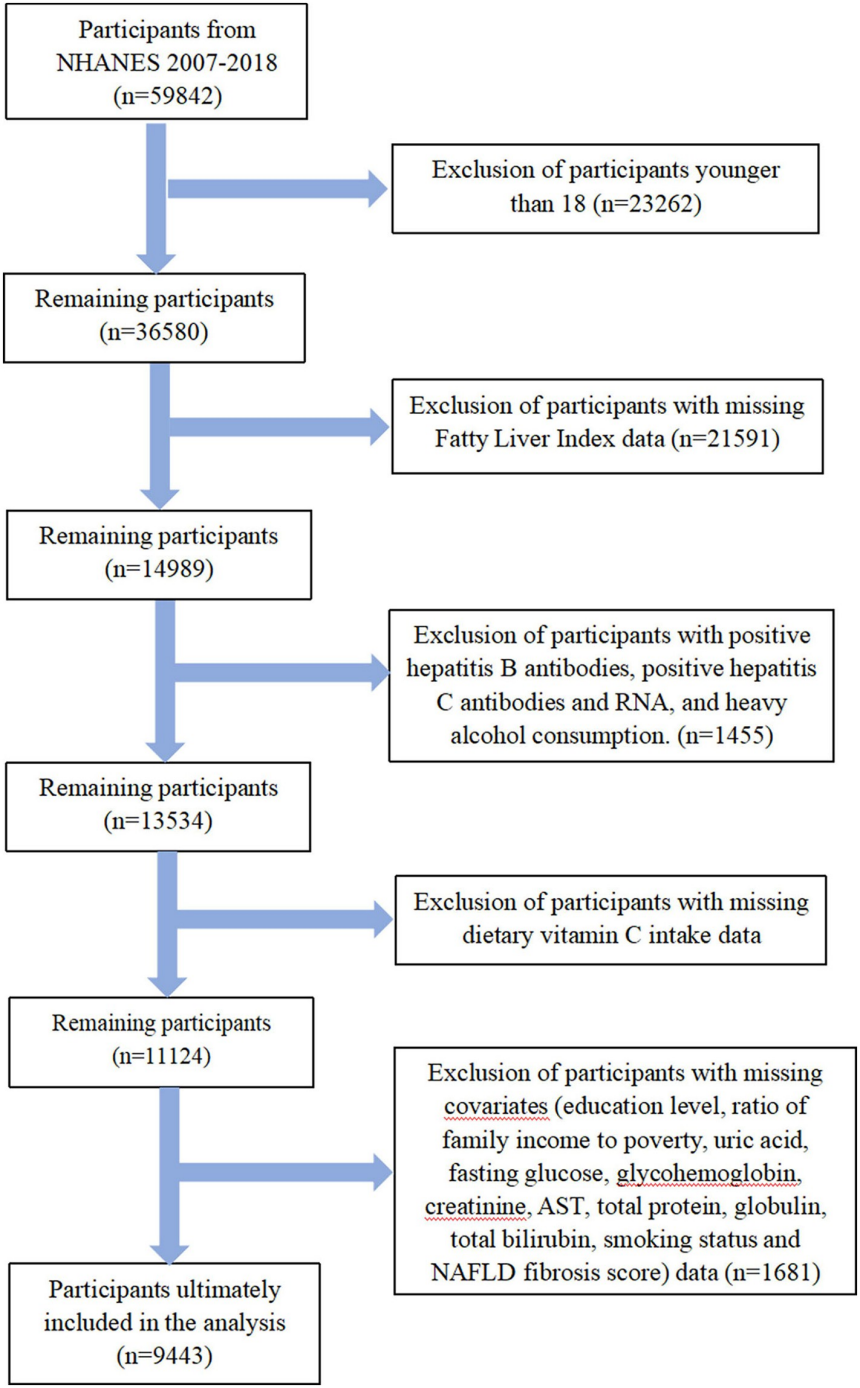
A flowchart showing the selection of study participants.

Data tagged as missing, refused, and did not know on the NHANES website were treated as missing data and were excluded. Participants aged <18 years, positive for hepatitis B antibody, hepatitis C antibody and RNA, and heavy alcohol consumption (> 30 g/d for men, > 20 g/d for women) were excluded. Participants were manually excluded for missing fatty liver index (FLI), NAFLD fibrosis score (NFS), dietary vitamin C intake, and covariates. Ultimately, a total of 9,443 participants were included in the analysis. The flowchart of the inclusion and exclusion criteria is shown in Fig 1. All participants provided written informed consent, and approval from the NCHS Research Ethics Review Board was applicable to the study. Fig 1 summarizes the exclusion criteria in a flowchart.

### 2.2 Definition of NAFLD and AHF

The FLI is a widely used surrogate marker for the prediction of NAFLD risk and is recommended by the European guidelines for the management of NAFLD [18, 19]. This study used the FLI to define NAFLD, and participants with an FLI greater than or equal to 60 were considered to have NAFLD [20]. The NFS score is a non-diffusion system that can identify fiber degeneration in nonalcoholic fatty liver disease. In this study, NAFLD patients with an NFS> 0.676 were considered to have an AHF. The FLI and NFS formulas are as follows [20, 21]:

$$\begin{array}{l} \text{FLI}=\left({\text{e}}^{0.953\times \text{loge}\left(\text{TG}\right)+0.139\times \text{BMI}+0.718\times \text{loge}\left(\text{GGT}\right)+0.053\times \text{WC}-15.\text{745}}\right)/\\ \left(1+{\text{e}}^{0.953\times \text{loge}\left(\text{TG}\right)+0.139\times \text{BMI}+0.718\times \text{loge}\left(\text{GGT}\right)+0.053\times \text{WC}-15.745}\right)\times 100; \end{array}$$

$$\begin{array}{l} \text{NFS}=-1.675+0.037\times \text{age}+0.094\times \text{BMI}+1.13\times \\ \text{impaired}\;\text{fasting}\;\text{glycemia}\;\text{or}\;\text{diabetes}\;(\text{yes}=1,\text{no}=0)+\\ 0.99\times \text{AST/ALT}\;\text{ratio}-0.013\times \text{platelet}-0.66\times \text{albumin}. \end{array}$$

### 2.3 Dietary vitamin C and potassium intake

Dietary data regarding dietary VC intake and potassium intake were assessed by the two 24 h diet recall interviews in NHANES. The details of the NHANES dietary survey are described elsewhere [22].

### 2.4 Covariates

To construct the regression model and to exclude the effect of potential factors on the outcome, some covariates were included in this study. Demographic variables included age, gender, race, education, and ratio of family income to poverty. The behavioral characteristics included smoking status, work activities status, and recreational activities status. Dietary energy, protein, and alcohol intake were taken as the dietary characteristics. The related disease conditions included hypertension status and diabetes status. The biochemical indicators included uric acid, fasting glucose, glycohemoglobin, total cholesterol, HDL cholesterol, creatinine, triglycerides, GGT, AST, ALT, total protein, globulin, and total bilirubin.

Mexican American, Other Hispanic, Non-Hispanic white, Non-Hispanic black, and Other Race individuals were categorized as ethnic groups. There are three levels of education: lower than high school, high school, and above. The ratio of family income to poverty was classified as <1,1 to3, and > 3. Smoking status was categorized as never, former, and now. Participants’ work or leisure-time activities were evaluated using four levels, including No, Vigorous, Moderate, and Both. Participants with hypertensive medication or past/current diagnosis were diagnosed with hypertension. Diabetes status was classified as yes, no, impaired fasting blood glucose (IFG), and impaired glucose tolerance (IGT) based on hypoglycemic medication administration status, diabetes diagnosis, HbA1c, and FG glucose. Participant indicator data were provided by the NHANES laboratory. All covariate data in this study were obtained from the NHANES website (https://www.cdc.gov/nchs/nhanes/index.htm).

### 2.5 Statistical analysis

Continuous variables were reported as "mean +—standard deviation" or "median (interquartile spacing)" for the sake of characterizing the participants, while categorical variables were described using weighted percentages (%). It is described using the median (interquartile spacing) when the standard deviation of the continuous variables is greater than half of the mean. Note that since participants who consumed a large amount of dietary alcohol were excluded from this study with a median and interquartile spacing of 0, dietary alcohol intake was described using the “mean ± standard deviation". The statistical significance of categorical and continuous variables was evaluated using the χ2 test and the Kruskal‒Wallis test, respectively.

The link between dietary vitamin C intake and NAFLD and AHF was examined using multiple logistic regression analysis, and analytical models based on variables were built. In addition, the covariates included in the study were tested for interactions to explore the interactive factors associated with the results. The 95% confidence intervals were calculated in this study.

The threshold of statistical significance was set at 0.05 for each analysis. For data processing, the researchers used Empower Stats software (www.empowerstats.net, X&Y solutions, Inc. Boston, Massachusetts) and the statistical package R (The R Foundation; http://www.r-project.org; version 3.6.3).

## 3. Results

### 3.1 Baseline characteristics of participants

The basic characteristics of the participants in the study are shown in Table 1.

**Table 1:** Baseline characteristics of participants.

Characteristic	NAFLD		P-value
	No	Yes	
N	5222	4221	
Demographics			
Age (year), Median (Q1-Q3)	47.50 (33.00–64.00)	53.00 (39.00–65.00)	<0.001
Sex			<0.001
Male	2250 (43.09%)	2136 (50.60%)	
Female	2972 (56.91%)	2085 (49.40%)	
Race			<0.001
Mexican American	662 (12.68%)	750 (17.77%)	
Other Hispanic	538 (10.30%)	437 (10.35%)	
Non-Hispanic White	2261 (43.30%)	1941 (45.98%)	
Non-Hispanic Black	979 (18.75%)	818 (19.38%)	
Other Race—Including Multi-Racial	782 (14.98%)	275 (6.52%)	
Education level			<0.001
<High school	1048 (20.07%)	1079 (25.56%)	
High school	1109 (21.24%)	1013 (24.00%)	
>High school	3065 (58.69%)	2129 (50.44%)	
Ratio of family income to poverty			<0.001
< = 1	1020 (19.53%)	927 (21.96%)	
1–3	2146 (41.10%)	1927 (45.65%)	
>3	2056 (39.37%)	1367 (32.39%)	
Behavioral Characteristics			
Smoking status			<0.001
Never	3239 (62.03%)	2257 (53.47%)	
Now	879 (16.83%)	734 (17.39%)	
Former	1104 (21.14%)	1230 (29.14%)	
Work activities status			0.022
No	3085 (59.08%)	2362 (55.96%)	
Vigorous	186 (3.56%)	164 (3.89%)	
Moderate	1180 (22.60%)	1011 (23.95%)	
Both	771 (14.76%)	684 (16.20%)	
Recreational activities status			<0.001
No	2359 (45.17%)	2499 (59.20%)	
Vigorous	475 (9.10%)	217 (5.14%)	
Moderate	1483 (28.40%)	1091 (25.85%)	
Both	905 (17.33%)	414 (9.81%)	
Dietary Characteristics			
Dietary energy intake (kcal), Median (Q1-Q3)	3659.50 (2844.25–4737.00)	3779.00 (2879.00–4814.00)	0.029
Dietary protein intake (g),Median (Q1-Q3)	143.91 (109.73–188.50)	149.50 (111.44–194.87)	0.001
Dietary alcohol intake (g), Mean ± SD	5.28 ± 11.90	4.49 ± 11.55	0.001
Dietary vitamin C intake (mg), Median (Q1-Q3)	134.70 (66.20–236.40)	115.00 (54.50–213.30)	<0.001
Related disease conditions			
Hypertension status			<0.001
No	3489 (66.81%)	1860 (44.07%)	
Yes	1733 (33.19%)	2361 (55.93%)	
Diabetes status			<0.001
No	3813 (73.02%)	1874 (44.40%)	
Yes	639 (12.24%)	1440 (34.12%)	
IFG	392 (7.51%)	538 (12.75%)	
IGT	378 (7.24%)	369 (8.74%)	
Anthropometric measurements			
Waist Circumference (cm), Mean ± SD	89.20 ± 9.65	113.49 ± 13.08	<0.001
BMI (kg/m^2), Mean± SD	25.09 ± 3.57	34.73 ± 6.27	<0.001
Biochemical indicators			
Uric Acid (mg/dl), Mean± SD	5.05 ± 1.27	5.98 ± 1.42	<0.001
Fasting Glucose (mg/dl), Mean± SD	102.24 ± 25.53	119.85 ± 43.50	<0.001
Glycohemoglobin (%), Mean± SD	5.57 ± 0.82	6.09 ± 1.32	<0.001
Creatinine (mg/dL), Median (Q1-Q3)	0.82 (0.69–0.98)	0.85 (0.72–1.02)	<0.001
GGT (IU/L), Median (Q1-Q3)	16.00 (12.00–22.00)	25.00 (18.00–39.00)	<0.001
Triglycerides (mg/dL), Median (Q1-Q3)	82.00 (60.00–113.00)	136.00 (96.00–192.00)	<0.001
Total cholesterol (mg/dL), Mean ± SD	187.71 ± 39.83	195.54 ± 42.19	<0.001
HDL cholesterol (mg/dL), Mean ± SD	58.05 ± 15.45	46.82 ± 12.13	<0.001
AST (u/l), Median (Q1-Q3)	22.00 (19.00–26.00)	23.00 (19.00–28.00)	<0.001
ALT(u/l), Median (Q1-Q3)	18.00 (15.00–24.00)	23.00 (18.00–32.00)	<0.001
Total Protein (g/dl), Mean ± SD	7.14 ± 0.45	7.15 ± 0.45	0.12
Globulin (g/dl), Mean ± SD	2.88 ± 0.44	3.01 ± 0.46	<0.001
Total Bilirubin (mg/dl), Median (Q1-Q3)	0.70 (0.50–0.90)	0.60 (0.50–0.80)	<0.001

Abbreviations: CDAI, Composite Dietary Antioxidant Index; NAFLD, Nonalcoholic Fatty Liver Disease; IFG, Impaired Fasting Glycemia; IGT, Impaired Glucose Tolerance; BMI, Body Mass Index; GGT, Gamma glutamyl transferase; AST, Aspartate Transaminase; ALT, Alanine Aminotransferase

*BMI was calculated as the body weight in kilograms divided by the square of the height in meters

P value: if it is a continuous variable, it is obtained by Kruskal Wallis rank sum test. If the theoretical number of counting variables is less than 10, it is obtained by Fisher exact probability test.

Among these participants, 4221 had NAFLD, and the other 5222 had no NAFLD. NAFLD is seen more commonly in males over the age of 50. Between the two groups, there was significant difference in race, education level or ratio of family income to poverty. Participants with NAFLD had higher dietary energy intake, waist circumference and BMI levels. Therefore, those with NAFLD are more likely to also have hypertension or obesity. In addition, biochemical indicators, except HDL cholesterol and total bilirubin, showed a higher number of people with NAFLD than those without NAFLD in all the indicators shown in Table 1.

### 3.2 Baseline characteristics with AHF as a stratified variable in the NAFLD population

AHF is an advanced clinical manifestation of NAFLD. Table 2 describes the baseline characteristics of the NAFLD population stratified as AHF. The number of people with NAFLD was 4221, which included 703 people with AHF and 3518 people who were not suffering from AHF. The majority of people with AHF are over 60 years of age, and the prevalence is similar for men and women. There were significant differences between the two groups in relation to race, education level and the ratio of household income to poverty, consistent with the results in Table 1. AHF is commonly seen in people with no work activities or recreational activities. Furthermore, people with AHF also have more hypertension or obesity and higher waist circumference and BMI than people without AHF.

**Table 2:** Baseline Characteristics with AHF as stratified variable in NAFLD population.

Characteristic	AHF		P-value
	No	Yes	
N	3518	703	
Demographics			
Age(year), Mean ± SD	49.45 ± 15.58	66.49 ± 11.32	<0.001
Sex			0.820
Male	1783 (50.68%)	353 (50.21%)	
Female	1735 (49.32%)	350 (49.79%)	
Race			<0.001
Mexican American	654 (18.59%)	96 (13.66%)	
Other Hispanic	368 (10.46%)	69 (9.82%)	
Non-Hispanic White	1587 (45.11%)	354 (50.36%)	
Non-Hispanic Black	661 (18.79%)	157 (22.33%)	
Other Race—Including Multi-Racial	248 (7.05%)	27 (3.84%)	
Education level			<0.001
<High school	856 (24.33%)	223 (31.72%)	
High school	848 (24.10%)	165 (23.47%)	
>High school	1814 (51.56%)	315 (44.81%)	
Ratio of family income to poverty			<0.001
< = 1	794 (22.57%)	133 (18.92%)	
1–3	1556 (44.23%)	371 (52.77%)	
>3	1168 (33.20%)	199 (28.31%)	
Behavioral Characteristics			
Smoking status			<0.001
Never	1904 (54.12%)	353 (50.21%)	
Now	678 (19.27%)	56 (7.97%)	
Former	936 (26.61%)	294 (41.82%)	
Work activities status			<0.001
No	1914 (54.41%)	448 (63.73%)	
Vigorous	150 (4.26%)	14 (1.99%)	
Moderate	841 (23.91%)	170 (24.18%)	
Both	613 (17.42%)	71 (10.10%)	
Recreational activities status			<0.001
No	1991 (56.59%)	508 (72.26%)	
Vigorous	203 (5.77%)	14 (1.99%)	
Moderate	933 (26.52%)	158 (22.48%)	
Both	391 (11.11%)	23 (3.27%)	
Dietary Characteristics			
Dietary energy intake (kcal), Median (Q1-Q3)	3847.00 (2927.25–4892.00)	3474.00(2635.00–4401.50)	<0.001
Dietary protein intake (g), Median (Q1-Q3)	152.12 (113.37–197.42)	152.12 (113.37–197.42)	<0.001
Dietary alcohol intake (g), Mean ± SD	4.82 ±11.94	2.85 ±9.21	<0.001
Dietary vitamin C intake (mg), Median (Q1-Q3)	116.75 (54.62–216.33)	107.70 (52.80–200.45)	0.063
Related disease conditions			
Hypertension status			<0.001
No	1716 (48.78%)	144 (20.48%)	
Yes	1802 (51.22%)	559 (79.52%)	
Diabetes status			<0.001
No	1813 (51.53%)	61 (8.68%)	
Yes	948 (26.95%)	492 (69.99%)	
IFG	404 (11.48%)	134 (19.06%)	
IGT	353 (10.03%)	16 (2.28%)	
Anthropometric measurements			
Waist Circumference (cm), Mean ± SD	111.63 ± 11.83	122.80 ± 14.93	<0.001
BMI (kg/m^2), Mean± SD	33.98 ± 5.62	38.48 ± 7.84	<0.001
Biochemical indicators			
Uric Acid (mg/dl), Mean± SD	5.91 ± 1.38	6.32 ± 1.55	<0.001
Fasting Glucose (mg/dl), Mean± SD	116.11 ± 41.40	138.57 ± 48.64	<0.001
Glycohemoglobin (%), Mean± SD	5.98 ± 1.27	6.66 ± 1.41	<0.001
Creatinine (mg/dL), Median (Q1-Q3)	0.84 (0.71–0.99)	0.94 (0.78–1.20)	<0.001
GGT (IU/L), Median (Q1-Q3)	26.00 (18.00–39.75)	23.00 (17.00–37.00)	<0.001
Triglycerides (mg/dL), Median (Q1-Q3)	139.00 (97.00–196.00)	125.00 (94.00–171.00)	<0.001
Total cholesterol (mg/dL), Mean ± SD	198.96 ± 42.00	178.42 ± 38.89	<0.001
HDL cholesterol (mg/dL), Mean ± SD	46.58 ± 11.91	47.99 ± 13.14	0.035
AST (u/l), Median (Q1-Q3)	23.00 (19.00–28.00)	23.00 (19.00–28.00)	0.509
ALT(u/l), Median (Q1-Q3)	23.00 (19.00–28.00)	20.00 (16.00–26.00)	<0.001
Total Protein (g/dl), Mean ± SD	7.17 ± 0.45	7.04 ± 0.46	<0.001
Globulin (g/dl), Mean ± SD	3.00 ± 0.45	3.06 ± 0.51	0.019
Total Bilirubin (mg/dl), Median (Q1-Q3)	0.60 (0.50–0.80)	0.60 (0.50–0.80)	0.727

Abbreviations: CDAI, Composite Dietary Antioxidant Index; NAFLD, Nonalcoholic Fatty Liver Disease; IFG, Impaired Fasting Glycemia; IGT, Impaired Glucose Tolerance; BMI, Body Mass Index; GGT, Gamma glutamyl transferase; AST, Aspartate Transaminase; ALT, Alanine Aminotransferase

*BMI was calculated as the body weight in kilograms divided by the square of the height in meters

P value: if it is a continuous variable, it is obtained by Kruskal Wallis rank sum test. If the theoretical number of counting variables is less than 10, it is obtained by Fisher exact probability test.

### 3.3 Association between dietary intake of vitamin C and NAFLD or AHF

**Fig 2:**
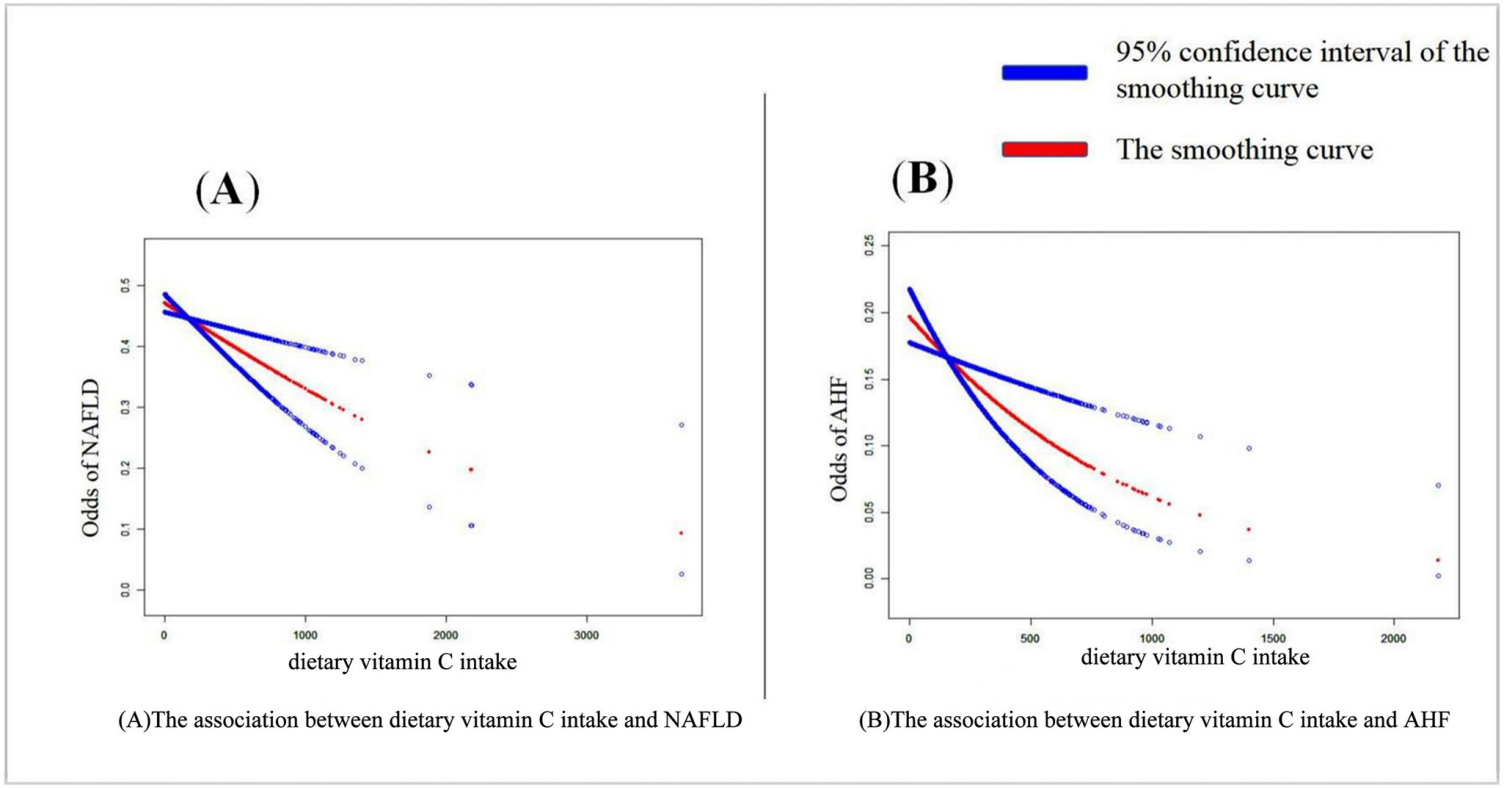
Smoothing curve fitting plot. (A) The association between dietary vitamin C intake and NAFLD. (B) The association between dietary vitamin C intake and AHF.

Table 3 shows the association between the three subgroups of dietary vitamin C intake levels and NAFLD or AHF. The low level of dietary vitamin C intake was used as a control to form a comparison with the prevalence of NAFLD in the middle-level group and the high-level group. First, the crude model is an unadjusted model that does not adjust for any variables. Table 3 adjusts a total of five models, with model 1 adjusting primarily for demographic variables, including age, ethnicity, education level, and ratio of poverty to income. The other models are adjusted for variables in turn, with the aim of deriving whether the results are stable across models. Adjusted model 5 is a fully adjusted model with all covariates adjusted. The group with the highest dietary intake of vitamin C had the lowest hazard ratio for NAFLD and AHF compared to the low-level group and was statistically significant (p < 0.05). In all six models, hazard ratios were stable in the three groups. In model 5, the group with highest dietary intake of vitamin C had a 28% lower risk of AHF (OR = 0.72, p = 0.0122). Smoothed curves adjusted for model 5 were used to visualize the association between dietary intake of vitamin C and NAFLD or AHF, and the results are shown in Fig 2. The results show a negative association between dietary intake of vitamin C levels and the prevalence of NAFLD and AHF.

**Table 3:** Association between dietary intake of vitamin C and NAFLD or AHF.

Models	NAFLD		AHF	
	OR (95%CI)	P-value	OR (95%CI)	P-value
Crude model				
Dietary intake of vitamin C				
Low	Ref.		Ref.	
Middle	0.79 (0.72, 0.87)	<0.0001	1.03 (0.84, 1.25)	0.7935
High	0.70 (0.63, 0.77)	<0.0001	0.86 (0.71, 1.06)	0.1546
Adjusted model 1				
Dietary intake of vitamin C				
Low	Ref.		Ref.	
Middle	0.80 (0.73, 0.89)	<0.0001	0.84 (0.67, 1.05)	0.1183
High	0.70 (0.63, 0.77)	<0.0001	0.72 (0.57, 0.91)	0.005
Adjusted model 2				
Dietary intake of vitamin C				
Low	Ref.		Ref.	
Middle	0.83 (0.75, 0.92)	0.0003	0.83 (0.67, 1.04)	0.1015
High	0.75 (0.67, 0.83)	<0.0001	0.72 (0.57, 0.91)	0.0052
Adjusted model 3				
Dietary intake of vitamin C				
Low	Ref.		Ref.	
Middle	0.81 (0.73, 0.90)	<0.0001	0.83 (0.66, 1.03)	0.0966
High	0.71 (0.64, 0.80)	<0.0001	0.70 (0.55, 0.89)	0.0032
Adjusted model 4				
Dietary intake of vitamin C				
Low	Ref.		Ref.	
Middle	0.81 (0.73, 0.90)	0.0002	0.79 (0.62, 1.00)	0.05
High	0.74 (0.66, 0.83)	<0.0001	0.69 (0.54, 0.89)	0.0042
Adjusted model 5				
Dietary intake of vitamin C				
Low	Ref.		Ref.	
Middle	0.82 (0.73, 0.93)	0.002	0.80 (0.62, 1.02)	0.0668
High	0.78 (0.69, 0.89)	0.0002	0.72 (0.56, 0.93)	0.0122

Crude model was not adjusted

Adjusted model 1 adjusted for age, race, education level, ratio of family income to poverty

Adjusted model 2 adjusted for model 1 + smoking status, work activities status, recreational activities status

Adjusted model 3 adjusted for model 2 + dietary energy intake, dietary protein intake, Dietary alcohol intake

Adjusted model 4 adjusted for model 3 + hypertension status, diabetes status

Adjusted model 5 adjusted for model 4 + uric acid, fasting glucose, glycohemoglobin, total cholesterol, HDL cholesterol, creatinine

### 3.4 Potassium affects the association between dietary intake of vitamin C and NAFLD

Table 4 shows that dietary vitamin C intake levels were divided into three groups, and in all three models, the OR was significantly lower in the T3 group than in the T1 group. Moreover, potassium levels were also divided into three groups. In all models within the T1 group, the OR was significantly higher in the high potassium group than in the low potassium group. In all models within group T2, the group with middle levels of potassium had the lowest OR and the lowest risk of NAFLD. In addition, of all the models within group T3, the high-level potassium group had the lowest OR (p<0.05), with a significant statistical significance.

**Table 4:** Potassium affects the association between dietary intake of vitamin C and NAFLD.

Exposure	T1		T2		T3		P interaction
	OR (95%CI)	P-value	OR (95%CI)	P-value	OR (95%CI)	P-value	
Crude model							0.0009
Potassium tertile							
Low	Ref.		0.87 (0.74, 1.02)	0.0829	0.84 (0.67, 1.04)	0.1070	
Middle	1.09 (0.93, 1.28)	0.2719	0.80 (0.69, 0.93)	0.0033	0.78 (0.67, 0.92)	0.0023	
High	1.49 (1.20, 1.86)	0.0003	0.91 (0.78, 1.06)	0.2345	0.73 (0.64, 0.83)	<0.0001	
Adjusted model I							0.0038
Potassium tertile							
Low	Ref.		0.87 (0.74, 1.02)	0.0919	0.85 (0.68, 1.06)	0.1416	
Middle	1.04 (0.88, 1.22)	0.6578	0.81 (0.70, 0.94)	0.0058	0.78 (0.66, 0.91)	0.0022	
High	1.34 (1.07, 1.68)	0.0099	0.86 (0.73, 1.02)	0.0840	0.69 (0.60, 0.79)	<0.0001	
Adjusted model II							0.0284
Potassium tertile							
Low	Ref.		0.94 (0.78, 1.15)	0.5603	0.89 (0.68, 1.16)	0.3844	
Middle	1.04 (0.85, 1.27)	0.7198	0.81 (0.67, 0.97)	0.0253	0.91 (0.75, 1.11)	0.3713	
High	1.27(0.95,1.69)	0.1088	0.83(0.66,1.04)	0.1019	0.74 (0.60, 0.90)	0.0033	

Crude model was not adjusted

Adjusted model 1 adjusted for age, race, education level, ratio of family income to poverty

Adjusted model 2 adjusted for model 1 + uric acid, fasting glucose, glycohemoglobin, total cholesterol, HDL cholesterol, creatinine, smoking status, work activities status, recreational activities status, dietary energy intake, dietary protein intake, dietary alcohol intake

## 4. Discussion

The results from this cross-sectional study showed that there was a negative correlation between nutrition C and NAFLD and AHF. Additionally, it has been found that potassium and vitamin C work together to reduce NAFLD.

To our knowledge, this is the first cross-sectional investigation to examine the influence of dietary potassium intake on the relationship between vitamin C consumption and both NAFLD and AHF. In terms of dietary risk factors, NAFLD is more likely to result from excessive strength consumption than micronutrient intake. [23, 24], but at present, the exact mechanism by which vitamin C reduces the prevalence of NAFLD is unclear and may involve three possible mechanisms. First, vitamin C may protect the liver by promoting cholesterol excretion [25]. Second, vitamin C has strong reducibility, which can inhibit oxidative stress and thus regulate lipid metabolism [26]. Third, through intestinal microbes and the intestinal-hepatic axis, vitamin C supports NAFLD patients’ liver characteristics and other associated metabolic parameters [8, 11].

Previous studies have found that taking more vitamin C, which can retard the development of hepatic fibrosis [27], is related to a lower possibility of NAFLD [28]. Our results supported those of Jie Wei and colleagues, who found a negative correlation between dietary vitamin C intake and NAFLD in middle-aged and older persons, especially in the nonobese male population [10]. Additionally, oral vitamin C supplementation can improve liver fitness in NAFLD patients, according to a randomized medical trial [11]. A cross-sectional study with 9254 participants demonstrated an inverse correlation between serum VC levels and hepatic fibrosis in terms of the link between vitamin C and AHF [9]. An animal experiment indicated that a medium dose of vitamin C was beneficial for NAFLD therapy, a low dose of vitamin C prevented the development of NAFLD, and a high dose of vitamin C for NAFLD management was risky. The viable mechanism is that HFD-induced iron overload probably leads to vitamin C autoxidation and in turn generates excessive H2O2 [29].

Potassium ions, which affect organs such as the heart, kidney and bone [16], are essential minerals for human health. In addition, potassium ions are both an anti-apoptotic agent and a pro-apoptotic agent [30, 31] which can effectively maintain the function and integrity of red blood cells by maintaining the biological energy and antioxidant enzyme activity of red blood cells, reducing the accumulation of malondialdehyde (MDA) and potassium ions, and inhibiting the content of free radicals [32]. Potassium deficiency is a potential risk factor for NAFLD [12–14, 32]. Potassium may be involved in the development of NAFLD by causing insulin deficiency [12, 13]. Hyperglycemia can lead to fatty degeneration by increasing synthesis of free fatty acids and lipid accumulation in liver tissues [15, 33–35]. Previous research has observed that the blood potassium level of NAFLD sufferers is lower than that of healthy people [15]. A cross-sectional study of middle-aged and elderly people in China found that hypokalemia was significantly related to the prevalence of NAFLD [17]. In an Italian study, there was a negative correlation between serum potassium levels and the histological severity of NAFLD in children [36]. However, a Korean study found that it may be influenced by eating habits and confounding factors, and K has nothing to do with NAFLD [37].

In addition, potassium ions can stabilize ascorbic acid free radicals [38]. Vitamin C may control the active transport of potassium ions in some tissues and allow a rapid diffusion of potassium into the cell [39, 40]. However, the mechanism of the relationship between potassium and vitamin C is still unclear.

Our research has some limitations. First, due to the cross-sectional design, we were unable to demonstrate causality or directionality. Even after multiple adjustments, the results can still be influenced by unmeasured variables. Second, because the study population was limited to residents of the United States, it must be taken into account when extrapolating to other populations. As a result, we need multicenter, controlled trials to validate our findings.

## 5. Conclusions

Overall, our results find out that vitamin C are negatively related with the threat of NAFLD and AHF amongst US adults. And the interaction of potassium on the association between vitamin C and NAFLD may be of great significance to the clinical use of drugs for the prevention of NAFLD.
